# Retinal Vascular Autoregulation during Phase IV of the Valsalva Maneuver: An Optical Coherence Tomography Angiography Study in Healthy Chinese Adults

**DOI:** 10.3389/fphys.2017.00553

**Published:** 2017-07-28

**Authors:** Yuan Zong, Huan Xu, Jian Yu, Chunhui Jiang, Xiangmei Kong, Yi He, Xinghuai Sun

**Affiliations:** ^1^Department of Ophthalmology and Vision Science, Eye and ENT Hospital, Fudan University Shanghai, China; ^2^Key Laboratory of Myopia of State Health Ministry and Key Laboratory of Visual Impairment and Restoration of Shanghai Shanghai, China; ^3^Department of Ophthalmology, Fifth People's Hospital of Shanghai Shanghai, China; ^4^The Key Laboratory on Adaptive Optics, Chinese Academy of Sciences Chengdu, China; ^5^The Laboratory on Adaptive Optics, Institute of Optics and Electronics, Chinese Academy of Sciences Chengdu, China

**Keywords:** valsalva maneuver, retinal vessel density, autoregulation, optical coherence tomography angiography, blood pressure

## Abstract

The impairment of retinal vascular autoregulation can be an early manifestation of many systemic and ocular diseases. Therefore, quantifying retinal vascular autoregulation in a non-invasive manner is very important. This study evaluated the effects of a Valsalva maneuver (VM)-induced blood pressure increases on retinal vascular autoregulation. Parafoveal and peripapillary retinal vessel density were measured with optical coherence tomography angiography before (baseline) and 5 s after each subject completed a VM (Phase IV [VM-IV]). Hemodynamic parameters and intraocular pressure (IOP) were examined. Blood pressure (systolic, diastolic, and mean arterial) and ocular perfusion pressure significantly increased during VM-IV, but IOP and heart rate (HR) did not change. The VM-induced blood pressure overshoot significantly decreased parafoveal (8.43%) and peripapillary (1.57%) perfused retinal vessel density (both *P* < 0.001). The response in the parafoveal region was greater than that in the peripapillary region (*P* < 0.001), and was age-dependent (*r* = 0.201, *P* < 0.05). Foveal avascular zone area detectable with OCTA significantly increased from baseline by 6.63% during VM-IV (*P* < 0.05). Autoregulatory responses to a VM did not show gender-related differences in either retinal region. The autoregulation of retinal vessels may vary in different regions of the fundus. Optical coherence tomography angiography could be a useful method for evaluating the autoregulation of the retinal vascular system.

## Introduction

Retinal vessel autoregulation is a complex homeostatic process that keeps blood flow constant during changes in blood pressure (BP; Robinson et al., 1986) and blood gas concentrations (most notably oxygen and carbon dioxide; Fallon et al., 1985). The pathogenic mechanisms of many systemic and ocular diseases, including chronic or malignant hypertension (Baumbach and Heistad, 1988; Immink et al., 2004), diabetes (Kohner et al., 1995), and primary open-angle glaucoma (Grunwald et al., 1984), have been linked to impaired autoregulation. Therefore, quantifying vascular autoregulation in a non-invasive manner is of great value for understanding, diagnosing, and treating retinal diseases associated with vascular dysregulation.

The Valsalva maneuver (VM) is performed by moderately exhaling against a closed airway. The VM is frequently performed during daily activities, including heavy lifting, vomiting, straining, forceful coughing, and sneezing. Compared with other methods for changing BP, the VM is a simple way to transiently increase BP (Reinhard et al., 2001). For this reason, the VM has been used to evaluate vessel autoregulation in many studies (Reinhard et al., 2001; Castro et al., 2014).

The VM has four phases based on the BP responses evoked (Novak, 2011). Phase I begins with an initial deep breath and lasts for 2–4 s. During this phase BP is transiently increased because of aortic compression caused by an intrathoracic pressure elevation. Phase II begins with forced exhalation. During this phase, BP initially decreases and then recovers back to or above baseline levels. Phase III begins with VM release and is characterized by a brief BP decrease. Phase IV begins at the end of Phase III, lasts for 10–20 s, and is characterized by an overshoot of arterial pressure.

The retinal vascular system is regulated automatically in response to variations in systemic BP or local oxygen tension (Dumskyj et al., 1996; Rassam et al., 1996). Because the retinal vasculature can be easily visualized and imaged in a non-invasive way, it provides an optimal model in which to study vascular autoregulation. (Robinson et al. (1986), using laser Doppler velocimetry and retinal vascular diameter measurements, demonstrated that the retina can maintain constant ocular perfusion during an increase in BP. However, partly because of the limitation of the technique, only the larger vessels were measured in their study. However, a more recent study demonstrated that capillaries are the first vessels to react and are responsible for >80% of the changes in cerebral blood flow (Hall et al., 2014). Recently, Jia et al. (2012a) quantified retinal circulation at the capillary level using high-speed optical coherence tomography angiography (OCTA) and a split-spectrum amplitude-decorrelation angiography algorithm.

The recent development of OCTA allows for quantitative evaluation of changes in retinal perfused vessel density. However, patients are required to be still during measurements to avoid introducing motion artifacts into OCTA images. Therefore, patients must fix their head in the instrument and fixate on an internal target for several seconds. Unfortunately, the transient BP elevation that occurs in VM Phase II often causes subjects to involuntary shake while intentionally straining. Therefore, we chose to obtain OCTA measurements of retinal perfused vessel density during VM Phase IV (VM-IV). More specifically, this study examined the effect of BP changes on the retinal microcirculation during VM-IV in healthy subjects.

## Materials and methods

This study protocol was reviewed and approved by the institutional review board of the Eye, Ear, Nose, and Throat (EENT) Hospital of Fudan University (Shanghai, China). All study conduct adhered to the tenets of the Declaration of Helsinki and written informed consent was obtained from all subjects.

### Study subjects

Healthy subjects who visited the EENT Hospital of Fudan University for a routine health screening were asked to participate in this study. Only the right eye of each subject was included in analyses. The inclusion criteria were: a best-corrected visual acuity (BCVA) of at least 16/20; no history of elevated intraocular pressure (IOP; < 21 mmHg); an axial length (AL) of 21–25 mm; and a spherical equivalent (SE) between −3 and + 1 D. The exclusion criteria were: a history of systemic disease, such as hypertension, diabetes mellitus, or cardiopulmonary insufficiency; the use of systemic or topical medication within the preceding 1 month; a history of intraocular disease, ocular surgery, or trauma; clinically relevant media opacities; and an inability to correctly perform a VM.

### Study examinations

All study subjects underwent a thorough ophthalmologic examination, including slit-lamp biomicroscopy, an undilated fundus examination (by direct ophthalmoscopy), and measurement of refractive error (with a Canon RK-5 Autorefractor Keratometer [Canon Inc. Ltd, Tokyo, Japan]), BCVA, IOP [with a Topcon CT-80A Computerized Tonometer (Topcon, Tokyo, Japan)], and AL (by optical biometry with an IOLMaster; Carl Zeiss AG, Jena, Germany). The SE of the refractive error was used in data analyses and was defined as the spherical error plus one-half of the cylindrical error.

The subjects were asked to sit and breathe normally for 20 min. The baseline OCTA scans were then performed with a spectral domain system (RTVue-XR Avanti; Optovue, Fremont, CA, USA; software version 2015.1.0.90) (Jia et al., 2012b; Yu et al., 2015), by a single observer. At the same time, baseline HR and systemic BP were measured using a semi-automated oscillometric BP recorder (NX-8102, Medical Device Corporation, Guang Zhou, China). Mean arterial pressure (MAP) was calculated as the diastolic blood pressure (DBP) plus one-third of the difference between the diastolic and systolic blood pressure (SBP). Ocular perfusion pressure (OPP) was calculated as two-thirds of the MAP minus the IOP.

### Valsalva maneuver

After the baseline data were collected, the subjects were asked to rest briefly (about 5 min), then were taught to do a modified VM, as previously described (Li et al., 2016). Subjects were asked to take a deep breath and then to forcefully blow it out against a closed glottis while squeezing their nose between an index finger and thumb. After 15 s, the subjects were asked to breathe normally. As previously demonstrated, after ending the VM, a sudden decrease in intrathoracic pressure causes BP to briefly fall (Phase III) for 1–2 s (Novak, 2011; Perry et al., 2014; Wada et al., 2014). To ensure that BP, HR, and retinal blood flow were measured at the same time during VM-IV, OCTA imaging was commenced 5 s after VM release. The perifoveal and peripapillary regions were scanned in a randomized order, selected with Microsoft Excel 2010 (Microsoft Corporation, Redmond, Washington, USA). Because a semi-automated oscillometric BP recorder was used for the BP and HR measurements, and 5–8 s are required for the cuff to create the necessary pressure, BP and HR were measured immediately after VM release. After a 1-h rest, subjects again performed a VM, and IOP was measured 5 s after VM release.

### Optical coherence tomography angiography

All OCT angiography images were obtained using a commercially-available spectral-domain OCT system, as described in Section Study Examinations. *En face* retinal angiograms were automatically generated using flow signal projection from the internal limiting membrane to the retinal pigment epithelium. Macular and optic nerve head data were acquired over a 6.0 × 6.0 mm and a 4.5 × 4.5 mm area, respectively. The software automatically calculated the perfused vessel density in the parafovea (annulus with an outer diameter of 3 mm and an inner diameter of 1 mm) peripapillary area (a 700-μm wide elliptical annulus extending outward from the optic disc boundary) and foveal avascular zone (FAZ) area.

### Analyses

Data are presented as mean ± standard deviation. Systemic parameters, IOP, FAZ, and retinal perfused vessel density before and after subjects performed a VM were compared using paired *t*-tests. The correlation between the independent variables age, sex, baseline BP, variation in BP, variation in OPP, and variation in MAP during VM-IV, with perfused vessel density response to VM as the dependent variable were analyzed using a linear regression model. All analyses were performed using SPSS statistical software (version 20.0, SPSS, Inc., Chicago, IL, USA) and statistical significance was defined as *p* < 0.05.

## Results

### Baseline subject characteristics

A total of 109 healthy volunteers (42 men, 67 women) were included in this study. Mean subject age was 44.91 ± 10.10 years (range: 19–61 years). Mean SD was −0.23 ± 0.98 D (range: −3.0–1.0 D), mean AL was 23.14 ± 0.78 mm (range: 21.51–24.84 mm), and mean baseline IOP was 12.62 ± 2.45 mmHg (range: 9–21 mmHg).

### Response to valsalva maneuver

#### Heart rate and pressure

Measurements of BP, HR, MAP, IOP, and OPP before and during VM-IV are summarized in Table 1. Briefly, SBP, DBP, MAP, and OPP significantly increased over baseline values during VM-IV (All *p* < 0.001). However, IOP (*p* = 0.411) and HR (*p* = 0.304) did not significantly change.

**Table 1:** Blood pressure, heart rate, intraocular pressure, and ocular perfusion pressure at baseline and during Phase IV of the Valsalva maneuver.

	**Baseline**	**Valsalva (phase IV)**	**% Change**	P^*^
SBP (mmHg)	121.61 ± 20.10	129.11 ± 19.65	6.94 ± 11.42	**<0.001**
DBP (mmHg)	76.63 ± 12.07	81.75 ± 13.40	7.24 ± 11.88	**<0.001**
MAP (mmHg)	91.81 ± 14.32	97.71 ± 14.61	6.98 ± 9.88	**<0.001**
IOP (mmHg)	12.62 ± 2.45	12.45 ± 2.35	−1.19 ± 15.16	0.411
OPP (mmHg)	48.77 ± 9.36	52.90 ± 9.66	9.47 ± 13.43	**<0.001**
HR (bpm)	74.89 ± 10.82	74.21 ± 12.61	−0.52 ± 9.33	0.304

*Data are presented as mean ± standard deviation (n = 109 subjects)*.

*SBP, systolic blood pressure; DBP, diastolic blood pressure; MAP, mean arterial pressure; IOP, intraocular pressure; OPP, ocular perfusion pressure; HR, heart rate*.

* *Statistical values were compared with paired samples t-test*.

*P-values in bold are statistically significant at P < 0.05*.

#### Retinal perfused vessel density

**Figure 1:**
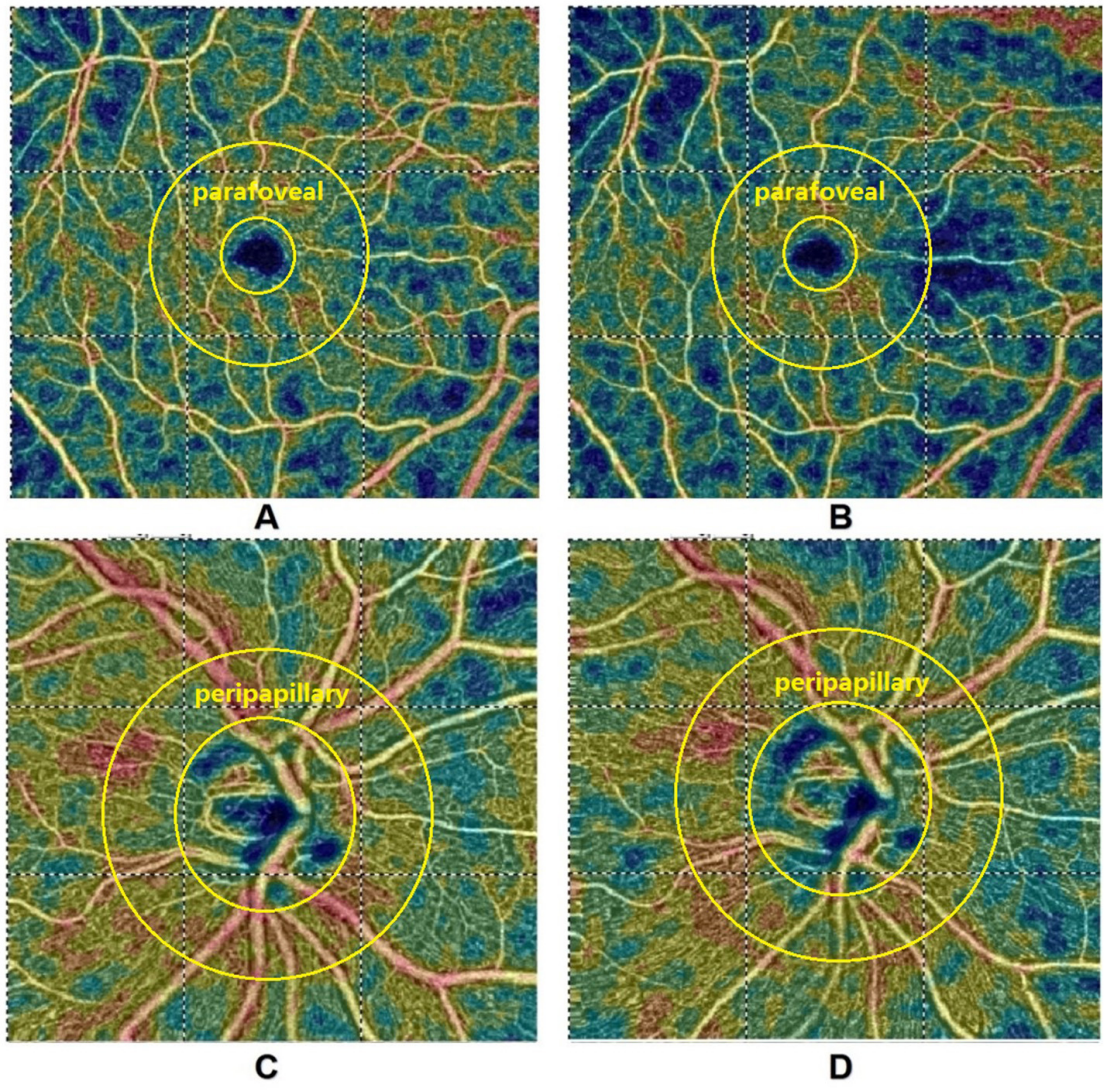
Optical coherence tomography angiographic images of the parafoveal **(A,B)** and peripapillary **(C,D)** regions at baseline **(A,C)** and during Phase IV **(B,D)** of the Valsalva maneuver. Parafoveal and peripapillary retinal perfused vessel density decreased by 10.2 and 3.6%, respectively. Diastolic blood pressure and systolic blood pressure increased by 1.2 and 10.4%, respectively.

Table 2 summarizes perfused retinal vessel density changes from baseline that occurred during VM-IV. Parafoveal and peripapillary retinal perfused vessel density significantly decreased by 8.43% ± 9.72% (*p* < 0.001) and 1.57% ± 3.98% (*p* < 0.001), respectively (Figure 1). Additionally, the FAZ area detectable with OCTA increased significantly during VM-IV by 6.63% ± 17.64% (*p* = 0.001).

**Table 2:** Perfused vessel density and foveal avascular zone area at baseline and during Phase IV of the Valsalva maneuver.

	**Baseline**	**Valsalva (Phase IV)**	**Change**	**% change**	P^*^
**PERFUSED VESSEL DENSITY**					
Parafovea	53.97 ± 4.10	48.41 ± 4.78	−4.69 ± 5.20	−8.43 ± 9.72	**<0.001**
Peripapillary	61.42 ± 2.51	60.43 ± 2.86	−0.99 ± 2.43	−1.57 ± 3.98	**<0.001**
FAZ area	0.38 ± 0.12	0.40 ± 0.13	0.02 ± 0.06	6.63 ± 17.64	**0.001**

*Data are presented as mean ± standard deviation (n = 109 subjects). FAZ, foveal avascular zone*.

* *Statistical values were compared with paired samples t-test*.

*P-values in bold are statistically significant at P < 0.05*.

Changes in perfused vessel density during VM-IV were also observed in different fundus regions. Retinal perfused vessel density decreased significantly more in the parafoveal region than in the peripapillary region (*p* < 0.001, Table 3). Gender did not significantly influence perfused vessel density in either the parafoveal (male: 7.76%, female: 8.86%; *p* = 0.295) or peripapillary (male: 1.80%, female: 1.42%; *p* = 0.566) region (Table 4).

**Table 3:** Difference in Valsalva maneuver response between the parafoveal and peripapillary regions.

	**Parafoveal**	**Peripapillary**	P^*^
Valsalva response	−4.69 ± 5.20	−0.99 ± 2.43	**<0.001**
Valsalva response, %	−8.43 ± 9.72	−1.57 ± 3.98	**<0.001**

*Data are presented as mean ± standard deviation (n = 109 subjects)*.

* *Statistical values were compared with paired samples t-test*.

*P-values in bold are statistically significant at P < 0.05*.

**Table 4:** Changes in retinal perfused vessel density in men and women that occurred during Phase IV of the Valsalva maneuver.

	**Men**	**Women**	P^*^
Parafoveal	−7.76 ± 10.64	−8.86 ± 9.12	0.295
Peripapillary	−1.80 ± 4.08	−1.42 ± 3.93	0.566

*Results are presented as mean ± standard deviation (n = 109 subjects)*.

* *Statistical values were compared with paired samples t-test*.

**Figure 2:**
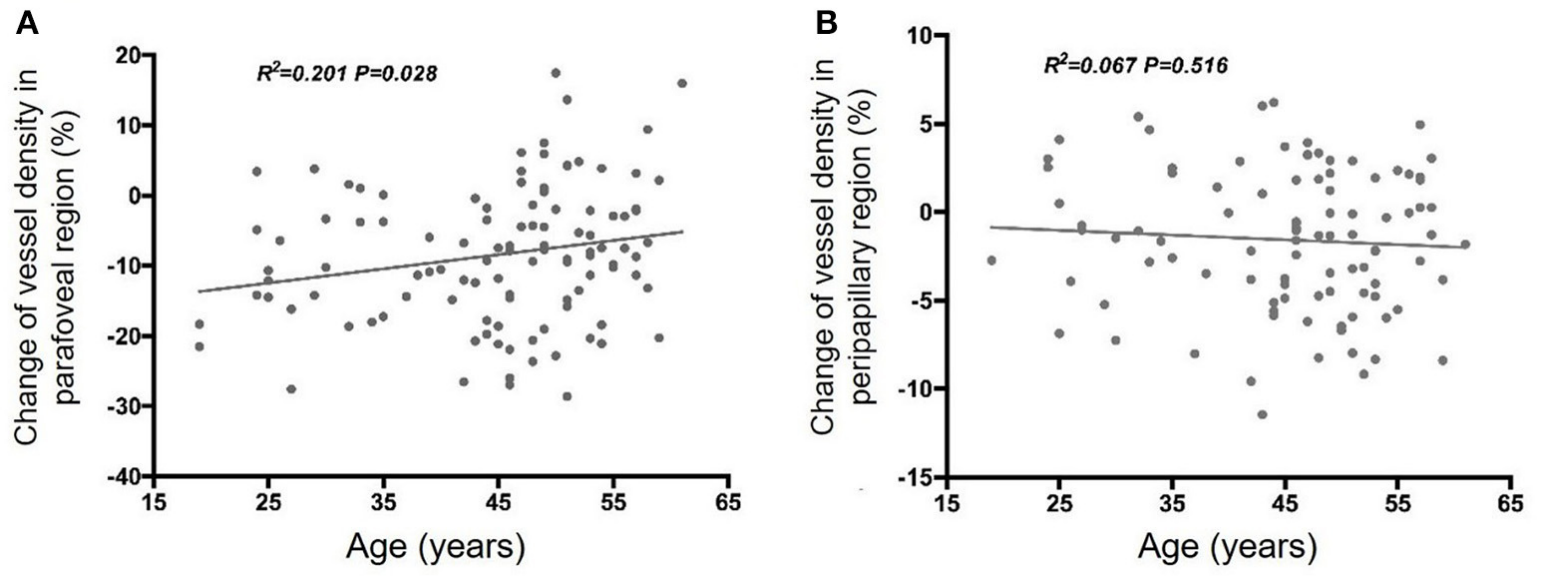
Correlation between age and Valsalva maneuver-induced response of retinal perfused vessel density in the parafoveal **(A)** and peripapillary **(B)** regions.

Linear regression analyses revealed a significant negative correlation between age and the parafoveal perfused vessel density response during VM-IV (β = −0.210, *p* = 0.028; Figure 2A). In contrast, peripapillary perfused vessel density changes during VM-IV were not significantly correlated with subject age (β = 0.067, *p* = 0.516; Figure 2B). The variation in the parafoveal and the peripapillary perfused vessel density were not correlated with sex, baseline BP, or the variations in DBP, SBP, OPP, or MAP during VM-IV (all *p* > 0.05).

## Discussion

In the current study, we assessed retinal vascular autoregulation in response to a VM-induced BP elevation in a group of healthy Chinese subjects. As expected, SBP, DBP, MAP, and OPP significantly increased from baseline during VM-IV. Furthermore, OCTA revealed a significant reduction in retinal perfused vessel density in both the parafoveal and peripapillary regions during VM-IV. A corresponding increase in the FAZ area detectable with OCTA was also observed. Interestingly, the reduction in retinal perfused vessel density was much greater in the parafoveal area than in the peripapillary area, and the magnitude of the parafoveal perfused vessel density response was negatively correlated with age. To the best of our knowledge, this is the first study to evaluate retinal microvascular autoregulation in response to VM-induced BP changes during VM-IV.

Previous studies have been conducted to examine retinal vascular changes in response to BP variations. These studies used pharmacological interventions [e.g., infusion of sympathomimetics (Rassam et al., 1995)] or physiological methods [e.g., isometric exercises (Dumskyj et al., 1996), the cold pressor test (Nagaoka et al., 2002), and position changes (Boltz et al., 2013)] to induce BP changes. However, these methods are all somewhat cumbersome and are not regarded as routine clinical examinations. In contrast, our study used the VM, a non-invasive, sensitive, and widely-used clinical test. The VM is also a standard stimuli used to investigate human blood flow autoregulation (Novak, 2011; Bohr et al., 2015).

The OPP increased from baseline by approximately 10% during VM-IV. This change led to a significant reduction in parafoveal and peripapillary retinal perfused vessel density in healthy subjects. This finding is consistent with the fact that the retinal vasculature constricts when OPP is elevated to keep blood flow constant. These findings are in agreement with previous studies by other investigators. Schmidl et al. (2012) found that autoregulation kept ocular nerve head blood flow constant until OPP increased from baseline by 80% or more. Jeppesen et al. (2007) and Movaffaghy et al. (1998) concluded that autoregulatory retinal vessel contraction or an increase in vascular resistance keeps retinal blood flow constant during acute, isometric exercise-induced elevations in OPP. Unfortunately, these studies only examined one segment of a retinal arteriole in the optic disc region. Hemodynamic parameters were then analyzed using a retinal vessel analyser or Laser Doppler flowmeter. Therefore, their measurements were limited by their local nature and by reduced measurement repeatability. Our study did not have these limitations because OCTA has a high repeatability (Yu et al., 2015) and produces high-resolution images of the entire retinal circulatory network, including larger vessels and their surrounding capillaries. Additionally, OCTA can quantify average regional retinal perfused vessel density (Jia et al., 2012a; Yu et al., 2015) and has the sensitivity to evaluate retinal vessel autoregulation under different conditions (Pechauer et al., 2015; Xu et al., 2016).

The mechanisms underlying retinal vessel autoregulation in response to systemic BP changes remain unclear. During VM-IV, BP is elevated via sympathetic nervous system stimulation. This nervous system stimulation does not directly affect retinal vessels because the retinal vasculature lacks autonomic innervation (Alm, 1992). Therefore, autoregulation is achieved via myogenic and metabolic factor interactions. A myogenic response occurs when an intraluminal pressure increases results in a pressure-dependent membrane depolarization in arterial muscle cells. This depolarization causes a reduction in the arterial diameter with the formation of 20-hydroxyeicosatetraenoic acid, a potent vasoconstrictor (Harder et al., 2011). Cole and Welsh (2011) demonstrated that the activation of myosin light chain kinase and myosin light chain phosphatase was also important in modulating pressure-induced myogenic variations. Retinal capillaries also play a vital role in retinal vessel autoregulation. Capillary lumen diameter is changed when pericytes that surround capillaries contract or dilate in a similar manner as retinal vascular smooth muscle cells (Anderson, 1996). Pericytes contraction is evoked by vasoactive factors, including endothelin-1, angiotensin II, norepinephrine, GABA antagonists, and adenosine triphosphate (Matsugi et al., 1997; Kawamura et al., 2003; Peppiatt et al., 2006; Markhotina et al., 2007). Unfortunately, the effect of the VM on molecular vasomotor signals remains unknown in retinal vessels.

We observed a greater reduction in retinal perfused vessel density in the parafoveal region than in the peripapillary region during VM-IV. This finding is in agreement with our previous study, which showed that the retinal vessels' response to hyperoxia was lower in the peripapillary region than in the parafoveal region (Xu et al., 2016). We do not yet understand why these two regional responses differ, but the following might explain. Jeppesen et al. (Jeppesen et al., 2007) reported that the change in the retinal arteriole diameter in response to an increase in BP correlated negatively with the baseline vessel diameter. This might explain the different changing of vessel density at different region of the fundus, as the parafoveal region just contains small vessels or capillaries, and in contrast, the peripapillary region not only embraces a dense network of capillaries but also four principal intraretinal arteries and secondary arterioles. Second, the surface nerve-fiber layer in the peripapillary region is supplied by both the retinal arterioles and the cilioretinal artery branches from the posterior ciliary arteries (Sugiyama et al., 1994; Hayreh, 2001), whereas the foveal region is fully supplied by the retinal artery system. A previous study by Schmidl et al. (2012) demonstrated that the changes in the choroidal circulation were smaller than those in the retina after BP was increased by squatting. Therefore, the differences in vessel size and blood supply might contribute to this result. Further structural and biomechanical studies are needed to better understand differences in diameter changes in parafoveal and peripapillary vessels.

Interestingly, we identified a negative correlation between subject age and the change in parafoveal vessel density during VM-IV. This relationship was not found in the peripapillary region, perhaps because of regional differences in retinal vessel structure and function. Nevertheless, our results demonstrate an age-related reduction in retinal vessel autoregulatory ability. In agreement, Jeppesen et al. (2004) also observed an age-dependent decrease in the retinal arteriolar myogenic response. These findings suggest normal, age-related changes should be considered when evaluating retinal autoregulatory dysfunction associated with diseases, including glaucoma, diabetic retinopathy, and arterial hypertension.

Unfortunately, we found no significant association between the percentage change in vessel density (including in the parafoveal and peripapillary regions) and the change in BP. The following factors might explain this result. First, although the increase in BP induced by VM-IV caused a reduction in the average vessel density, the standard deviation for this change was relatively large. This indicates that the capacity for retinal vascular autoregulation (the BP response induced by VM-IV) differs among healthy individuals, so the percentage change in vessel density per unit increase in BP is unclear. Second, OCTA images only show the retinal vessel density, and not the retinal blood flow. Consistent with our results, Jeppesen et al. (2007) reported that the retinal diameter response was not significantly correlated with the change in MAP.

### Limitations

Our study had several limitations. First, IOP and perfused retinal vessel density could not be simultaneously monitored during VM-IV. Thus, subjects were required to perform the VM twice; once to measure IOP changes and once to measure retinal vascular changes. While BP was measured only during the first VM procedure, this likely increased measurement error and reduced OPP measurement accuracy. However, the current and prior studies (Polska et al., 2007) have shown that acute BP elevations (induced via VM and isometric exercise, respectively) only minimally affect IOP; and the assumption was made that BP was similarly modulated during the second VM procedure when IOP measurements were made because VM was described as a reproducible method (Baldwa and Ewing, 1977; Schmetterer et al., 1998; Pstras et al., 2016). This indicates that any introduced measurement error did not heavily influence our results. Second, ECG gating and continuous BP monitoring were not used in this study (Novak, 2011). Third, OCTA cannot detect red blood cells moving at velocities outside its detection range. A future study, using continuous BP monitoring and other more-advanced methods to visualize retinal vessels, may provide more insight into retinal microvasculature autoregulation.

## Conclusion

This study combines a classic method for elevating BP with a novel technique to evaluate regional retinal microvasculature autoregulation. Our OCTA measurements show that BP changes during VM-IV induce a significant reduction in retinal perfused vessel density in both the parafoveal and peripapillary regions. Furthermore, changes in retinal perfused vessel density are negatively correlated with age in the parafoveal region.

## Author contributions

CJ and XK designed of the work, modified the paper; YZ and HX did the data acquisition and wrote the paper; JY and YH did the data analysis; XS modified the paper.

### Conflict of interest statement

The authors declare that the research was conducted in the absence of any commercial or financial relationships that could be construed as a potential conflict of interest.
